# Infectious Ulcerative Stomatitis

**Published:** 1899-08

**Authors:** John S. Marshall

**Affiliations:** Chicago, Ills.


### Infectious Ulcerative Stomatitis.

BY DR. JOHN S. MARSHALL, CHICAGO, ILLS.

Abstract of paper read before the Section of Stomatology, American Medical Association, Columbus, June, 1899.

Stomatitis is an inflammation of the mucous membrane of the mouth. All inflammatory conditions which involve the gums, the inner surfaces of the cheeks, the lips and the tongue are usually included under the general term, stomatitis.

The affections which are thus included are, with few exceptions, confined to the period of infancy and childhood. Adults seldom suffer from these affections, except as manifestations of some other morbid condition.

The forms of inflammation of the mouth which are most common are *stomatitis simplex*, *stomatitis catarrhalis*, *stomatitis aphthosa*, *stomatitis parasitica* and *stomatitis ulcerosa*.

A clinical study of the inflammatory affections of the mucous membrane of the mouth will reveal in certain features a close resemblance to the inflammatory affections as they appear in the skin, while in other points they will present features which are common to the inflammatory conditions of the mucous membrane in general.

*Stomatitis Simplex* is the mildest form of stomatitis, and is generally expressed in a more or less intense redness of the surface of the mucous membrane of the cheeks, the lips and the gums, in the form of rose-red elevated patches, and is due to a localized hyperemia of the parts.

It is usually found in infants and young children, and it is generally associated with some form of gastric or intestinal derangement. It rarely persists for more than a few days, and as a rule disappears with the subsidence of the gastric or intestinal disturbance. It is rarely seen in the adult. Occasionally it may persist and gradually pass into a severer type of the disease known as stomatitis catarrhalis.

*Stomatitis Catrrhalis*, or follicular stomatitis, is an affection which is often a symptomatic expression of some more grave constitutional malady, such as inflammatory conditions of the alimentary and respiratory tracts, etc., though it may be induced by the local irritation of erupting teeth. It is common among children living in unsanitary districts, but rarely seen in adults. The disease is characterized by a diffuse inflammation, spreading upon the hard palate as streaks and patches. The papillæ of the tongue are affected, many of them appearing as prominent tubercles. The mucous glands become swollen and prominent, and can be readily felt by passing the finger over the surface of the membrane. Later they appear as "grayish or grayish-red elevations of the surface, surrounded by a reddened areola."—Ziegler. Tiny crypts sometimes develop, which are caused by the retention of the secretions. Swelling of the tongue, lips, cheeks and gums often occurs, accompanied by fetor of the breath, heat and dryness of the mouth, followed by an excessive salivary secresion. In the severer cases the gums become soft and spongy, and bleed upon the slightest provocation. Fissures form at the angles of the mouth and upon the lips, with exudation and the formation of crusts. The constitutional symptoms are fever, diarrhea, thirst, loss of appetite and sleeplessness.

*Stomatitis Aphthosa*, or canker sore mouth, is by some authorities thought to be a peculiar form of stomatitis catarrhalis, for the reason that the aphthous patches occur upon the oral mucous membrane during a catarrhal condition. The disease is most common in sickly children during the periods of dentition, and occasionally later in life in those who are debilitated from illness or debauchery. It often occurs in women during menstruation, in pregnancy and during the puerperal periods. Occasionally it is associated with pheumonia, bronchitis, diphtheria, exanthematous diseases, ague, whooping-cough and tonsilitis.

The disease appears in the form of a small white or yellowish-white patches upon the mucous membrane of the edges of the tongue and at the gingivo-buccal fold of the lips and the cheeks. They are slightly elevated above the surrounding membrane, and are exceedingly sensitive. The patches are surrounded by a more or less inflamed zone, and having a tendency to spread and coalesce forming larger patches.

The constitutional symptoms rarely exceed a slight elevation of temperature, loss of appetite, thirst and irritability.

*Stomatitis Parasitica* or *Thrush*, is a parasitic or mycotic affection, generally found in the mouths of infants and little children. The fungus which produce the disease is known as the "thrush funges" or *oidium albacans*, which grows upon and between the layers of the epithelium, but develops most rapidly upon the squamous type of epithelium.

It is most common among ill-fed and bottle-fed children, and is due to unwholesome milk and improperly cleansed nursing bottles. The disease is infectious, and in foundling and maternity hospitals it spreads rapidly from child to child, if great care is not exercised in sterilizing the food and the feeding apparatus. The growth of the organism is favored by an abnormal acidity of the oral secretions, a debiliated condition of the system and bad hygienic and sanitary surroundings.

The disease appears as small, white, elevated patches upon the inside of the lips, cheeks, sides of the tongue and just within the corners of the mouth. The mouth is dry and feverish, and the salivary secretion is scanty. After two or three days the patches assume a curdy or soft appearance. This curdy surface, the " thrush film," is easily peeled off, and leaves a denuded surface, which bleeds easily. It is soon covered again, however, by a new growth of the parasitic organism. The denuded surface is exceedingly sensitive, and renders feeling very painful, so that it is with great difficulty that little children can take food.

The extension of the disease to the pharynx, œsophagus, tonsils, hard palate and air passages is not an uncommon course for it to pursue. The constitutional symptoms are elevation of temperature, disorders of the stomach and intestines, with vomiting and diarrhea. The excreta from the bowels are often greenish in color, mixed with curdy masses of milk, and are often exceedingly acid, causing excoriation of the anus, buttock, perineum, etc. The disease is sometimes seen in adults who have suffered from prolonged and wasting diseases like typhoid fever, tuberculosis, etc. In little children the disease sometimes terminates fatally from exhaustion and inanition.

*Stomatitis Ulcerosa.* This is a much more serious condition than any of the forms of stomatitis which have been mentioned, and is more often seen by the dental surgeon and the stomatologist, for the reason that it most frequestly occurs in children from five to ten years of age, who are suffering from debility induced by disease, deficient food or unhealthy surroundings. It is occasionally seen in adults who have been debilitated from disease or debauchery. Local injuries and irritations from diseased teeth, and chronic poisoning by mercury, phosphorus, lead and copper (Ziegler), may also be causative factors in the production of the disease.

The disease is first manifest in the margins of the gums, usually in the anterior part of the mouth, by swelling, redness, loosening of the gums from around the necks of the teeth, accompanied by tenderness or pain. The loosened festoons and margins later become very much swollen and congested, partially covering the crowns of the teeth; finally they take on a purple hue, soften and slough away as yellow masses, leaving an irregular ulcerating surface. In the more serious cases the progress of the ulceration is rapid, and often extends to the deeper tissues, involving the border of the alveolar process, which may become necrosed, resulting in the loss of considerable portions of bone and even of several teeth. The breath is fetid, the salivary secretions are increased, and often mixed with pus and blood. The lymphatics often become swollen and tender. The cheeks and lips opposite the ulcerated portions of the gingivæ sometimes take on inflammatory conditions and ulceration, accompanied with considerable swelling. Food is taken with difficulty.

The disease usually appears as an acute affection, but occasionally it becomes chronic and lasts for months, in which case it becomes troublesome to cure.—(Tomes.)

The constitutional symptoms in the milder cases which involve only a limited area of ulceration are slight febrile disturbance and loss of appetite, which lasts for a few days and subsides upon the healing of the ulcerations. In the more severe cases in which the ulceration is extensive, involving the periosteum and the bone, the temperature may run quite high and not subside for several days. The high fever is accompanied by thirst, loss of appetite and great restlessness. The disease sometimes resembles gangrenous stomatitis, or *gangrenea oris.*

With this brief resume of the history of the various forms of stomatitis which are generally recognized, and which I have introduced for the purpose of comparison, I desire to call your attention to another form of ulcerative stomatitis which sometimes follows injuries to the gums from extraction of teeth, abrasions from hard foods or the vigorous use of the tooth brush, etc., and for which I propose the term *Stomatitis Ulcerosa Nocens*, or *Infectious Ulcerative Stomatitis.*

The clinical characteristics of this form of the disease are the formation of ulcers at some point of injury, which at first appear in nowise different from the ordinary form of a localized ulcerative stomatitis, but which after the lapse of twenty-four to forty-eight hours begins to spread rapidly along the margins of the gingivæ in all directions, involving both jaws and sometimes extending to the hard palate and the floor of the mouth. The margins of the gums assume a general ulcerative condition, accompanied by swelling, redness and considerable congestion of the parts, which bleed easily. Later they become covered with a dirty-white or yellowish-white pellicle or membrane—somewhat resembling the thrush film—which sloughs off after a day or two, destroying the festoons and leaving a ragged surface. The denuded surface is very red and covered with coarse granulations, which bleed upon the slightest provocation. The gums are loosened from the necks of the teeth, and the borders of the alveolar processes are exposed. Pus mixed with blood exudes from the inflamed tissue about the necks of the teeth. The breath and excretions are very fetid, and salivation is profuse. In these respects the symptoms resemble mercurial ptyalism. The ulcerated surfaces are exceedingly sensitive, and motions of the tongue and lips on this account are quite painful. Food is taken with difficulty.

Accompanying the local manifestations there is a general febrile condition, temperature ranging from 100° to 101° F., thirst, loss of appetite and general malaise, sleeplessness and irritability of temper.

In illustration of the above clinical features of the disease, the following cases are introduced:

Case 1.—Mr. A., American, aged twenty-one years, clerk, was referred for special treatment by Dr. P. I. Lawrence, Chicago.

*History.*—This gentleman had an abscessed lower molar of the right side extracted, which had caused considerable swelling of the jaw. The gum tissue had been somewhat lacerated upon the lingual side in the effort to remove the offending root. Two days later he returned with the injured gum ulcerated, the ulceration spreading to the adjoining teeth. Antiseptics had been used to cleanse the mouth, the alveolus irrigated and dressed, and a listerine mouth-wash prescribed. The disease, however, spread so rapidly that in forty-eight hours the gums of the entire lower jaw were involved, and it had attacked the anterior portion of the upper jaw. This was the condition when the case first came under my notice.

Diligent inquiry could not discover any constitutional conditions, like syphilis, mercurial or lead poisoning, etc., which would account for the presence of the disease. Had, however, recently been ill for a couple of weeks from a mild attack of la grippe.

*Treatment.*—The treatment consisted of first cleansing the mouth by irrigating it with a saturated solution of boric acid, followed by a 50 percent solution of twelve volume hydrogen peroxide in water sprayed into the mouth, and between the approximal spaces between the teeth. The mouth was again irrigated with boric acid solution, to remove all the debris and the foam caused by the use of the peroxide. After which the gums were carefully dried and protected with rolls of bibulous paper and the ulcerated surfaces swabbed with a 10 percent solution of zinc chlorid.

The patient was furnished with a bulb atomizer, and instructed to spray the mouth every two hours with 25 percent listerine solution.

This line of treatment was followed every day for a week, except the application of the zinc chlorid, which did not seem necessary after the third day, as marked improvement took place from this date. The case was discharged cured at the end of ten days.

The only constitutional treatment was a saline cathartic, which seemed to be indicated to relieve a tendency to constipation. The fact that local treatment alone, except that just indicated, was sufficient to control the case precludes the possibility of syphilitic infection being the cause of the affection.

Case 2.—This patient was a married man, aged thirty-four years, and of English birth, formerly a practicing dentist, but now an expert accountant.

*History.*—Patient states that he has been overworked of late, that his gums had been congested and bled when the teeth were brushed, and thinking that perhaps he had not been vigorous enough in the use of the tooth brush, bought a new one that was quite hard and gave them a most thorough brushing before retiring. Next morning his mouth was so greatly inflamed that he could not use the tooth brush or masticate his food, or even take a cup of hot coffee. For the next two days he tried to allay the inflammation with various soothing preparations, with no benefit. At this stage of the case he presented himself for examination and treatment.

Examination of the mouth revealed extensive ulceration of the margins of the gums of both jaws, with ulcerating streaks upon the roof of the mouth, extending from the region of the first molars on each side nearly to the median line, and looking as though they had been cauterized with silver nitrate. The ulcerations in all parts of the mouth were covered with the same dirty-white or yellowish-white film, and all of the other symptoms corresponding to those of Case 1. In Case 2, however, nearly every tooth in the mouth had a ring of salivary calculus encircling the cervix. This was, no doubt, the cause of the congested condition of the gums which induced the bleeding on brushing.

*Treatment* consisted of first cleansing the mouth, and then removing the salivary calculus. In all other respects the treatment was the same as in Case 1. He made a rapid recovery, and was discharged at the end of two weeks.

Case 3 was almost identical with Case 1. It originated from the same cause, namely, the extraction of an abscessed lower molar, followed by ulceration of the gingival wound, and extension of the ulcerative process to the gingival borders of both jaws. In this case, which occurred in a young Jew, twenty-four years of age, there was a clear history of syphilis, infection having taken place two years before. He had visited Hot Springs and taken a course of treatment, but had taken no mercury or iodides since his return, four months before.

The treatment prescribed in the other cases was followed in this, with the exception that after the third day of treatment, in consultation with his family physician, he was placed upon the usual course of treatment with the iodides. He rapidly improved under the local treatment from the first, and at the end of ten days all of the local symptoms had disappeared. From this I think the inference may be safely drawn that the local disease was not the result of his syphilitic condition, as it is hardly to be supposed that the constitutional effect of the iodides would be manifested in so short a period, while at the same time it was evident that the case was improving before the iodides were administered.

Neither can the first or third cases be fairly attributed to infection from unclean instruments, as I am sure that the greatest care was observed in both cases to prevent such a contingency. The explanation would rather, it seems to me, be that of auto-infection from the pus micro-organism of the alveolar abscess coming in contact with a freshly wounded surface of the gum or from some of the other pathogenic organisms which so constantly inhabit the mouths of even cleanly persons.

The second case was also, without doubt, due to auto-infection from the last-named causes, through the brushing and lacerating of the already inflamed gums, thus furnishing the only condition lacking before to establish an infectious inflammation, which, by reason of the debilitated condition of the system, it was unable to successfully resist.

The acute character of the symptoms and the rapid spreading of the ulceration from the initial point of injury seems to prove the infectious nature of the disease.

